# Advances in diagnosis and prediction for aggression of pure solid T1 lung cancer

**DOI:** 10.1093/pcmedi/pbad020

**Published:** 2023-08-17

**Authors:** Junhao Mu, Jing Huang, Min Ao, Weiyi Li, Li Jiang, Li Yang

**Affiliations:** Department of Respiratory and Critical Care Medicine, the First Affiliated Hospital of Chongqing Medical University, Chongqing 400016, China; Department of Respiratory and Critical Care Medicine, the First Affiliated Hospital of Chongqing Medical University, Chongqing 400016, China; Department of Respiratory and Critical Care Medicine, the First Affiliated Hospital of Chongqing Medical University, Chongqing 400016, China; Department of Respiratory and Critical Care Medicine, the First Affiliated Hospital of Chongqing Medical University, Chongqing 400016, China; Department of Respiratory and Critical Care Medicine, the First Affiliated Hospital of Chongqing Medical University, Chongqing 400016, China; Department of Respiratory and Critical Care Medicine, the First Affiliated Hospital of Chongqing Medical University, Chongqing 400016, China

**Keywords:** solid, T1, lung cancer, aggression, prediction

## Abstract

A growing number of early-stage lung cancers presenting as malignant pulmonary nodules have been diagnosed because of the increased adoption of low-dose spiral computed tomography. But pure solid T1 lung cancer with ≤3 cm in the greatest dimension is not always at an early stage, despite its small size. This type of cancer can be highly aggressive and is associated with pathological involvement, metastasis, postoperative relapse, and even death. However, it is easily misdiagnosed or delay diagnosed in clinics and thus poses a serious threat to human health. The percentage of nodal or extrathoracic metastases has been reported to be >20% in T1 lung cancer. As such, understanding and identifying the aggressive characteristics of pure solid T1 lung cancer is crucial for prevention, diagnosis, and therapeutic strategies, and beneficial to improving the prognosis. With the widespread of lung cancer screening, these highly invasive pure solid T1 lung cancer will become the main advanced lung cancer in future. However, there is limited information regarding precision medicine on how to identify these “early-stage” aggressive lung cancers. To provide clinicians with new insights into early recognition and intervention of the highly invasive pure solid T1 lung cancer, this review summarizes its clinical characteristics, imaging, pathology, gene alterations, immune microenvironment, multi-omics, and current techniques for diagnosis and prediction.

## Introduction

Lung cancer is the leading cause of cancer-related death around the world.^1–3^ A growing number of early-stage lung cancers presenting as malignant pulmonary nodules have been diagnosed because of the increased adoption of low-dose spiral computed tomography.^4^ But pure solid T1 (T1, ≤3 cm in the greatest dimension) lung cancer is not always early-stage lung cancer. A pure-solid nodule was defined as a consolidation tumour ratio equal to 1.0 and without a ground-glass opacity component on chest Computed Tomography (CT). It can exhibit highly aggressive behavior which is associated with pathological involvement, metastasis, postoperative relapse, and even death.^5,6^ It has been reported that the percentage of nodal or extrathoracic metastases is >20% in T1 lung cancer,^7–10^, i.e. some pure solid T1 lung cancer patients are diagnosed after the development of symptoms with metastatic sites. The aggressive behaviour may be linked to differences in cellular proliferation and invasion capabilities,^11–16^ is easy to misdiagnose or delay diagnosis in clinics and poses a serious threat to human health.

Understanding the demographic and biological characteristics of pure solid T1 lung cancer is critical for matching appropriate patients with individualized therapy strategies and forecasting prognoses. A proportion of pure-solid nodules reveal a pure solid appearance at the outset, while the remainder evolve from ground-glass nodules.^17,18^ A few studies have shown that more male than female patients have both pure solid and pathologic pure invasive tumours.^19–21^ Small solitary lung cancers which occur in males, younger patients, or locate in the main bronchus or left lung, or with histologic type as small cell lung cancer, or with undifferentiated type, tend to have extra-thoracic metastasis.^22^ However, there is limited information on how to identify patients with these “early-stage” lung cancers who present with aggressive characteristics. Therefore, this review focuses on summarizing the characteristics of the clinic, imaging, pathology, driver gene and single-cell multi-omics, machine learning, artificial intelligence (AI), and current methods for the diagnosis and prediction of highly invasive pure solid T1 lung cancer to improve early recognition and intervention for clinicians.

## Evaluating the aggressive characteristics of pure solid T1 lung cancer by chest CT images

Tumour size of lung cancer is associated with nodal upstaging, even in T1 diseases.^23^ Pure solid tumours may have a worse prognosis than part-solid tumours in stage IA patients according to the eighth edition tumor node metastasis (TNM) classification, and similar results were found for the T1a-1b and T1b-1c subgroups.^24^ Patients with a tumour size of T1b-1c (between 2 and 3 cm in size) exhibited more frequent nodal upstaging than those with a T1a-1b tumour size of ≤2 cm.^25,26^ Shin found that the prognosis of pure solid tumors remained worse, while the maximum tumour size of part solid tumours is larger than that of pure solid tumours.^27^ Hu *et al*. found that the incidence of extra-thoracic metastasis (M1b) in all small solitary lung cancer (≤2 cm) is 6.31% (692/10 968), and the incidence is elevated rapidly with an increase of the tumour size, from 0.98% in lesions ≤10 mm to 5.33% in lesions 11 to 20 mm in diameter. But the difference between solid T1 lung cancer and ground glass opacity (GGO) T1 lung cancer was not illustrated in their study.^22^ Tumour location at CT in the inner one-third of the lung, defined by concentric lines arising from the hilum, was adversely associated with survival and showed moderate inter-reader agreement.^28^ The central location may be associated with lymph node metastasis, but the tumour location seemed to have less impact on a GGO-predominant lesion.^29^ Density is regarded as a more important factor than location in evaluating the aggression of lung cancer. Nevertheless, it is difficult to identify the tumour density on High Resolution CT (HRCT) images, which complicates classification of a tumour area as solid or as a GGO component.^30^

## Pathological features are still an essential factor for distinguishing invasive pure solid T1 lung cancer

Solid lung cancer nodules are common presentations of various histological types, including adenocarcinoma, squamous cell carcinoma (SCC), and neuroendocrine tumours (NETs). These histopathological types have distinct molecular aggressive characteristics, different response patterns to systemic therapy, and varying prognoses.^31–33^ Adenocarcinoma is the most common histopathological type in non-small cell lung cancer (NSCLC) and is mostly prone to nodal upstaging in T1N0 disease.^24,27,34^ A collapsed lepidic component is detected as a solid component on HRCT images that usually represents a pathological invasive component.^35–37^ However, lung adenocarcinoma demonstrating lepidic growth (growth along alveolar walls) is a significantly favourable prognostic factor for pT1 NSCLC in multiple studies, despite showing a pure-solid appearance.^38,39^ However, pure-solid lung tumours presenting solid and micropapillary subtypes are frequently associated with a poorer prognosis.^40–42^ SCC used to be a representative histological type of centrally located lung cancer, but an increasing number of peripheral SCCs have been reported. A retrospective investigation found that peripheral-type SCC had a lower pathological stage, less lymphatic and vessel involvement, and fewer lymph node metastases, but there was no significant difference in overall survival compared to central-type SCC. In contrast, the present study showed that even small-sized SCCs in the peripheral lung may have malignant potential, such as a higher incidence of pleural, vascular, and lymphatic invasion.^31^ It was reported that the incidences of pleural invasion, vascular invasion, and lymphatic invasion were all significantly higher in SCC than in adenocarcinoma.^43–46^ But some studies found no difference in prognosis between SCC and solid adenocarcinoma.^31,47^ NETs of the lung with distinct clinical behavior constitute ∼20% of all primary lung tumours, including low-grade typical carcinoids, intermediate-grade atypical carcinoids, high-grade large cell neuroendocrine tumours, and small cell lung cancer. Histologic grade was the dominant driver of prognosis in patients with NETs. NETs appear as solitary tumours on CT, and their oncological behaviours are remarkably different.^48^ Another study reported that small cell lung cancer or with undifferentiated type tend to have extra-thoracic metastasis. Thus, pathological type is an essential factor for distinguishing the invasive T1 lung cancer.^22^

Visceral pleural invasion (VPI) may have a more significant association with poor prognosis in pure-solid tumours than in part-solid tumours. Some studies have suggested that cancer cells can spread through the pleural cavity and lymphatics to the mediastinal lymph nodes, and upstaging of the T-category by VPI in T1-sized NSCLCs may be more applicable for pure-solid tumours.^49^ VPI indicates poor prognosis and is defined as a T2 descriptor for T1-sized tumours.^50,51^ On the other hand, vascular invasion was identified as a poor-prognosis factor, even in stage IA NSCLC, because of a higher rate of distant metastasis.^31^ Therefore, evaluating the pathological VPI and vascular invasion is useful for predicting the invasiveness of T1 solid lung cancer.

## Different characteristics of molecular pathogenesis may drive invasiveness in the different stages of T1 lung cancer

According to the progression schema, gene alterations should be evenly accumulated along the entire progression of lung cancer. Epidermal Growth Factor Receptor (EGFR) -mutated tumours were evenly distributed along each progression step.^17,52^ In the T1c radiologically pure-solid lung adenocarcinoma subset, the EGFR-mutant group exhibited marginally lower 5-year Recurrence Free Survival (RFS) than the EGFR wild-type group.^17^ Furthermore, Kirsten Rat Sarcoma viral oncogene (KRAS) mutations are detected in 33% of Atypical Adenomatous Hyperplasia (AAH), 12% of carcinomas *in situ*, 8% of minimally invasive adenocarcinoma, and 0% of well-differentiated adenocarcinoma, which appear to decrease along the progression.^53^ Strikingly, Caucasians and smokers seemed to develop more rapidly progressive NSCLC, and the presence of a KRAS mutation may be responsible for the rapid progression of NSCLCs in Caucasian patients in some studies.^19^ Compared with subsolid nodule lung adenocarcinomas, solid nodule lung adenocarcinoma harboured higher somatic mutation counts, genomic alteration counts, and intratumour heterogeneity, and showed a lower incidence of EGFR mutation but a higher frequency of genes such as Tumor Protein P53 (TP53), AT-rich interactive domain-containing protein 1A (ARID1A), Phosphatidylinositol 4,5-bisphosphate 3-kinase catalytic subunit alpha isoform (PIK3CA), Recombinant Cyclin Dependent Kinase Inhibitor 2A (CDKN2A), and Black Rock Arts Foundation (BRAF) in terms of driver genes. In addition, they had a significantly higher number of pathway alterations, including p53, cell cycle, and phosphoinositide 3 kinase (PI3K), indicating that solid nodule lung adenocarcinomas had a less complex genomics architecture overall.^54^ However, the molecular mechanisms underlying this phenomenon are not yet clear.

Pathological nodal involvement was also thought to be a valuable prognostic factor in patients with pure-solid stage I NSCLC.^55^ Fusion mutation was a significant risk factor for lymph node metastasis among stage T1 NSCLC. In Shin's study, Anaplastic Lymphoma Kinase (ALK) rearrangement was correlated with more regional lymph node metastasis and unfavourable disease-free survival compared to ALK-negative patients in patients with completely resected stage IA lung adenocarcinoma.^56^ Similarly, ROS proto-oncogene 1 (ROS1) status was significantly associated with lymph node metastasis, and ROS1 positivity was found to be higher in patients at advanced node stages.^57^ Additionally, patients who possess the proto-oncogene tyrosine-protein kinase receptor Ret (RET) fusion gene tended to present with more N2 stage (54.5%) in small tumours (≤3 cm), significantly higher than the other lung adenocarcinomas without RET fusion (22.6%).^58^ Therefore, the fusion mutant protein may be a predictor for lymph node metastasis of solid T1 lung cancer.

## Multiomics sequencing analysis is an important technique to evaluate the invasiveness of T1 solid lung cancer and needs to be further developed

The presented studies that used multi-omics techniques to analyse the invasiveness of T1 lung cancer mainly focus on comparing the differences between solid T1 lung adenocarcinoma and T1 GGO lung adenocarcinoma. Adenocarcinomas exhibiting as solid nodules are often more aggressive and are characterized by a higher recurrence rate after resection.^59^ While multiple treatment modalities have substantially improved clinical outcomes for patients with adenocarcinoma, the treatment response can vary widely, ranging from long-term remission to rapid progression.^60,61^ Conventional bulk sequencing has uncovered abundant molecular aberrations that drive carcinogenesis and the progression of adenocarcinoma. More advanced techniques, such as single-cell RNA sequencing (scRNA-seq), are required to comprehensively decipher the underlying molecular mechanism and indicate the heterogeneity of adenocarcinoma.^62^ Tao conducted single-cell RNA sequencing on fresh surgical specimens obtained from eight resectable adenocarcinoma cases, of which four were identified as pure GGO and the remaining four as solid nodules on CT imaging. The results of the study indicated that the antitumour immunity mediated by natural killer (NK) and CD8^+^ T cells gradually weakened as adenocarcinoma progressed, while humoral immunity mediated by plasma B cells was more active in solid nodules. Moreover, stromal cells and M2 macrophages were found to promote the progression of adenocarcinoma. Through comprehensive analyses, they discovered dynamic changes in cellular components and intercellular interactions during the progression of adenocarcinoma. Lu *et al*. found that cancer cells derived from solid adenocarcinoma had a high-grade malignancy compared with those from GGO adenocarcinoma based on scRNA-seq results.^63^

The immune microenvironment was more active in solid nodule-associated adenocarcinomas than in GGO-associated adenocarcinomas, including more expression of immune-related genes, upregulation of immune pathways, more infiltration of immune cells, and an expanded TCR repertoire.^64^ The tumour immune microenvironment, especially tumour-infiltrating T cells, plays a pivotal role in the occurrence and development of tumours. Limited evidence has shown that the infiltration of tumor associated macrophages (TAMs) and Regulatory (Treg) cells and the expression of TGF-β are significantly higher in solid lung adenocarcinoma than in GGO-lung adenocarcinoma. The infiltration of the remaining immune cells, including CD8^+^ T cells, CD4^+^ T cells, CD103^+^ T cells, CD20^+^ B cells, and CD138^+^ plasma cells, did not seem to be significantly different in GGO-lung adenocarcinoma, although there was no significant difference in PD-L1 expression in the two cohorts.^52^ It is necessary to explore the differences in the immune microenvironment in highly aggressive pure solid T1 lung cancer in future. Multi-omics analysis has also revealed that solid-associated lung cancers are characterized by a more active metabolism and immune microenvironment, which may also be relevant to their aggressive clinical course.^65^ These findings highlight the importance of considering both molecular and immune factors in understanding the biology of solid T1 lung cancer and developing new treatment strategies, but the underlying mechanisms driving tumour progression and the intertumoural and intratumoural cellular and molecular heterogeneity in T1 solid lung cancer are limited.^66,67^

Analysing the spatial transcriptomic information of highly invasive lung cancer nodules is of the utmost importance for further understanding the mechanisms underlying metastasis. However, currently, there are no relevant reports available, primarily due to the limited availability of samples. The population with metastatic lung cancer is usually classified as locally advanced or advanced stages, where surgical resection is not recommended. Obtaining samples from these patients through non-surgical methods, such as non-surgical biopsies in medical departments, presents challenges as it yields a small sample size and makes it difficult to preserve the spatial structure, thus limiting the ability to accurately reflect the spatial architecture of the tumour. Despite these challenges, the direction of this research is highly promising and deserves attention. With advancements in detection and sampling techniques, we anticipate that valuable insights into the transcriptomic landscape of highly invasive lung cancer nodules and their metastatic mechanisms will be obtained.

The above information about prediction of aggressive characteristics has been summarized in Table 1.

**Table 1.** Prediction of aggressive characteristics in pure solid T1 lung cancer.

	Risk factors	Protective factors
Clinical characteristics	Younger patients, male, Caucasians, smokers	
Chest CT images	Density: pure solid Size (3–2 cm, >2–1 cm, >1–0 cm) Located in the main bronchus, left lung, the inner one-third of lung	Ground glass
Pathological features	Small cell lung cancer undifferentiated type, squamous cell carcinoma, solid adenocarcinoma; visceral pleural invasion, vascular invasion	Lepidic component
Molecular pathogenesis	Mutation of EGFR, KRAS, p53, cell cycle, and PI3K Fusion mutant protein: ALK rearrangement, ROS1 positivity, RET positivity Somatic mutation counts Genomic alteration counts Intratumour heterogeneity	
Multiomics sequencing	Weakened antitumour immunity Active humoral immunity Active metabolism and immune microenvironment	

## Advances in prediction for aggression of pure solid T1 lung cancer

Confirming the presence of pure solid lung cancer before an invasive procedure is challenging in clinical practice, especially in early stages. Several research findings indicated that iodine uptake in the arterial phase on contrast CT of primary lung cancer may be associated with some aspects of tumour histopathology, such as angiogenesis, differentiation grade, hypoxic cells, and tumour invasiveness.^68,69^ A low 3D-Iodine-related attenuation at the early phase of solid lung cancers has been significantly associated with pathological invasiveness and postoperative recurrence, as well as a higher TNM stage, pleural invasion, chest wall invasion, intrapulmonary metastasis, lymph node metastases, and poorer prognosis. These findings may help identify patients with early-stage lung cancer who have a higher risk of aggressive disease and may require more aggressive treatment strategies.^70^

On the other hand, the differences in malignant behaviour can be identified using maximum standardized uptake values (SUV) determined by F-18-fluorodeoxyglucose positron emission tomography/computed tomography (PET-CT).^71^ The SUVmax level correlated well with the histologic subtypes based on the International Association for the Study of Lung Cancer /American Thoracic Society / European Respiratory Society (IASLC/ATS/ETS) classification, even for cases of stage IA solid lung cancer.^72^ However, it should be noted that the accuracy of PET-CT for detecting lymph node metastasis is not always sufficient, particularly in regions where tuberculosis is endemic, due to the potential for false-positives caused by granulomatous inflammation.^73^

Meanwhile, several studies have demonstrated the potential of deep learning algorithms in predicting the aggressiveness and prognosis for lung cancer. Deep Cubical Nodule Transfer Learning Algorithm (CUBIT), a deep learning algorithm using transfer learning and 3D Convolutional Neural Networks (CNN) , can accurately predict lymphovascular invasion (LVI) or nodal involvement in T1 size NSCLC on CT images, which may be helpful in individualizing treatment. Tau developed a CNN for PET images to accurately designate the N category of previously untreated NSCLC patients.^74^ However, an integrated deep learning approach that combines multimodal imaging data with clinical data may be even more useful in predicting LVI or nodal involvement before surgery.^75^ Recently, we found that the deep learning method can accurately predict local or distant metastasis in patients with solid T1-stage lung cancer.^76^ However, high-quality mass data are needed to further develop and validate the prediction models to identfy the aggression of T1 solid lung cancer.

Liquid biopsy is a promising noninvasive alternative for cancer screening. carcinoembryonic antigen (CEA) has been identified as a predictor for mediastinal nodal metastasis in clinical stage IA NSCLC patients, and preoperative serum CEA levels can be used to predict tumour aggressiveness and recurrence in c-T1a lung cancer.^6^ Tsai also found that CEA alteration was associated with a significantly worse 5-year mortality rate in T1a-bN0M0 NSCLC patients.^77^ In recent years, circulating tumour DNA (ctDNA) has emerged as a promising liquid biopsy biomarker for noninvasive cancer screening and post-treatment surveillance.^78^ Due to the cell-free DNA (cfDNA) abundance of noncancerous origin coupled with its rapid metabolic rate, the concentration of ctDNA is extremely low, especially in operable early-stage cancers.^79^ Circulating tumor cells (CTCs) are one of the mainstays of liquid biopsy, which also includes circulating tumour DNA (ctDNA), cell-free RNA, extracellular vesicles, and tumour-educated platelets. CTCs are believed to be a precursor of metastasis, contributing to the high mortality rate associated with cancer.^80^ Furthermore, Ye *et al*. reported that circulating genetically abnormal cells (CACs) presented a significant diagnostic value in detecting lung cancer for patients with pulmonary nodules ≤10 mm.^81^ In their study, when the cutoff value of CACs was >2 mm, the sensitivity and specificity for lung cancer were 70.5% and 86.4%, respectively. Male, maximum solid nodule, maximum nodule located in upper lobe, and CACs >2 mm met the *P* < 0.10 criterion for inclusion in the multivariable models.^81^ With advancements in technology, future studies may be conducted to evaluate the efficacy of CACs in predicting metastasis of T1 solid lung cancer. Liquid biopsy is continuously improving, making it a promising tool for cancer diagnosis and monitoring.

**Figure 1.**
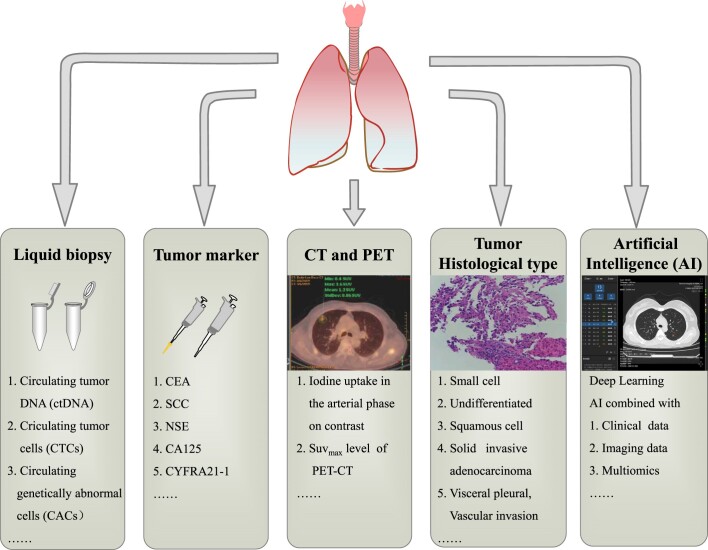
Prediction for aggression of pure solid T1 lung cancer.

To sum up, the current methods for prediction for the aggression of pure solid T1 lung cancer are shown in Fig. 1.

## Perspectives

Predicting oncological behaviours is important for surgical planning and aggressive surveillance of the tumour. In clinical and pathological T1N0-staged NSCLC, pure-solid tumours are associated with worse disease-free survival (DFS).^82,83^ The 8th edition of the TNM classification recommends that tumours with the same solid component size be further categorized into part-solid and pure-solid tumours and that they be considered separately due to the more malignant behaviour and poorer prognosis of pure-solid tumours. Larger pure-solid tumours may indicate the need for aggressive adjuvant or neoadjuvant therapy.^82^ However, the factors influencing tumour growth remain poorly defined, and in the absence of a clear relationship between time and tumour growth in NSCLCs, these factors will be key to identifying aggressive tumours that upstage early and need prompt resection. In the future, further exploration is needed for screening, diagnosis, treatment, and follow-up strategies for different early-stage lung cancers with varying degrees of invasiveness to achieve personalized optimal therapeutic effects.
